# Current-Induced Domain Wall Motion and Tilting in Perpendicularly Magnetized Racetracks

**DOI:** 10.1186/s11671-018-2655-6

**Published:** 2018-08-15

**Authors:** Dong Li, Baoshan Cui, Jijun Yun, Minzhang Chen, Xiaobin Guo, Kai Wu, Xu Zhang, Yupei Wang, Jian Mao, Yalu Zuo, Jianbo Wang, Li Xi

**Affiliations:** 1 Key Laboratory for Magnetism and Magnetic Materials of Ministry of Education & School of Physical Science and Technology, Lanzhou University, Lanzhou, 730000 People’s Republic of China; 2 Research Institute of Materials Science, Shanxi Normal University, Linfen, 041004 People’s Republic of China

**Keywords:** Spin-orbit torque, Perpendicular magnetic anisotropy, Dzyaloshinskii-Moriya interaction, Domain wall motion, Domain wall tilting

## Abstract

The influence of C insertion on Dzyaloshinskii–Moriya interaction (DMI) as well as current-induced domain wall (DW) motion (CIDWM) and tilting in Pt/Co/Ta racetracks is investigated via a magneto-optical Kerr microscope. The similar DMI strength for Pt/Co/Ta and Pt/Co/C/Ta samples reveals that DMI mainly comes from the Pt/Co interface. Fast DW velocity around tens of m/s with current density around several MA/cm^2^ is observed in Pt/Co/Ta. However, it needs double times larger current density to reach the same magnitude in Pt/Co/C/Ta, indicating DW velocity is related to the spin-orbit torque efficiency and pinning potential barrier. Moreover, in CIDWM, DW velocity is around 10^3^ times larger than that in field-induced DW motion (FIDWM) with current-generated effective field keeping the same magnitude as applied magnetic field, revealing that the current-generated Joule heating has an influence on DW motion. Interestingly, current-induced DW tilting phenomenon is observed, while this phenomenon is absent in FIDWM, demonstrating that the current-generated Oersted field may also play an essential role in DW tilting. These findings could provide some designing prospects to drive DW motion in SOT-based racetrack memories.

**Electronic supplementary material:**

The online version of this article (10.1186/s11671-018-2655-6) contains supplementary material, which is available to authorized users.

## Background

Current-induced magnetic domain wall motion (CIDWM) in racetracks has revealed a newly developing magnetic racetrack memory device [1, 2]. Owing to this promising prospect, lots of work have been carried out during the last few decades. CIDWM was firstly investigated in ferromagnets (FMs) with in-plane magnetic anisotropy and the spin-polarized current-generated spin transfer torque (STT) acts as the driving force [3, 4]. Afterwards, CIDWM was also realized in FMs with perpendicular magnetic anisotropy (PMA) [5, 6]. However, in some PMA materials, domain wall (DW) motion direction is opposite to the direction of the electron flow, which is contradictory to the prediction of STT [7, 8]. And much more works are found that DW motion is along the current direction in heavy metal (HM)/FM bilayer structures with PMA. It was demonstrated that HM-generated spin-orbit torques (SOTs) by spin Hall effect and/or Rashba effect together with the interfacial Dzyaloshinskii–Moriya interaction (DMI) due to the structural inversion asymmetry of FM are considered to drive a chiral DW motion along the current direction [9, 10]. Therefore, in order to enhance the efficiency of CIDWM, it requires the HM with large spin Hall angle (*θ*_*SH*_) to generate a larger torque to drive DW motion. Many efforts have been devoted to obtain a large *θ*_*SH*_ of HMs by varying the thickness of HM [11, 12], decorating the interface between HM and FM [13, 14], changing the crystallinity of HM [15], and even involving oxygen in HM [16]. Besides, some reports also achieve large effective *θ*_*SH*_ based on HM/FM/HM structures, in which two HM layers have opposite sign of *θ*_*SH*_ [17–19]. When a current passing through the two HM layers, the generated spin currents from two kinds of HM layers will work in concert to improve the SOT efficiency for decreasing the current density to switch the magnetization or drive the DW motion. Meanwhile, the DMI strength in this kind of trilayers may be different from the bilayers, since there are two interfacial interactions on both sides of the FM layer. It was found that DMI strength has a large influence on DW velocity when an extended collective coordinate model was proposed to explain the DW tilting behavior [20]. In addition, the DW tilting was also reported in GaMnAs micro-wires [20–22].

In our previous work, we have investigated the effect of inserting a C interlayer between Co and Ta on the anisotropy field, switching field, and SOT effective fields in Pt/Co/Ta structures with PMA [23]. The obtained magnetization switching current density is in the order of 10^6^ A/cm^2^ in both Pt/Co/Ta and Pt/Co/C/Ta devices. In this work, we investigate the current-induced DW motion and tilting behavior in these two samples and the influence of C insertion on DMI strength and DW velocity in micro-sized Pt/Co/Ta racetracks. We found a little change of the calculated DMI exchange constant (|*D*|), indicating that the DMI strength mainly comes from the contribution of the Pt/Co interface in Pt/Co/Ta and Pt/Co/C/Ta stacks. In field-induced DW motion, the measured DW velocity in Pt/Co/C/Ta is smaller than that in Pt/Co/Ta even under a large magnetic field, revealing that the pinning potential barrier has a great influence on DW motion. Besides, in CIDWM, a larger DW velocity is observed compared to that in field-induced motion with the same magnitude between the current generated effective field and applied magnetic field. It reveals that the current-generated Joule heating also affects the DW motion. More importantly, current-induced DW tilting phenomenon is observed in Pt/Co/Ta and Pt/Co/C/Ta stacks, which can be well explained by the current-generated Oersted field combined with spin Hall effective field.

## Methods

Two film stacks Ta(3)/Pt(5)/Co(0.6)/Ta(5) and Ta(3)/Pt(5)/Co(0.6)/C(2)/Ta(5) (thickness in nm) were deposited on corning glass substrates at room temperature by direct current magnetron sputtering with a base pressure below 4.0 × 10^−5^ Pa. The bottom 3 nm Ta is used as the seed layer, and the top Ta layer has an around 1.5 nm TaO_x_ capping layer due to the air exposure [17, 24]. Afterwards, the film stacks were patterned into 8.5-μm and 3.0-μm wide racetracks for Pt/Co/Ta and Pt/Co/C/Ta, respectively, using standard lithography and Ar-ion milling techniques to investigate CIDWM. Moreover, the 8.5-μm wide Hall bars patterned using the same techniques were used to measure the out-of-plane field (*H*_*z*_)-dependent anomalous Hall resistance (*R*_*Hall*_) at different in-plane bias fields (*H*_*x*_) along the current direction to achieve the spin Hall effective field (*H*_*SHE*_) and estimate the DMI strength as reported by Pai et al. [25]. In their report, the shift of *R*_*Hall*_-*H*_*z*_ loops at *H*_*x*_ can be well explained by a chiral Néel DW model. The shift was defined as *H*_*SHE*_, which can be used to quantify the SOT efficiency *χ* ≡ *H*_*SHE*_*/J* (*J* is the charge current density). The method was used to characterize the DMI strength and SOT efficiency in this work. Moreover, a magneto-optical Kerr microscope with polar Kerr effect was used to monitor the DW motion under the applied field or current pulse at room temperature.

## Results and Discussion

Based on the chiral Néel DW model, we firstly investigated the anomalous Hall loops under the in-plane bias field *H*_*x*_ to obtain DMI strength and SOT efficiency (see the Additional file 1). The obtained DMI effective field (*H*_*DMI*_) for Pt/Co/Ta and Pt/Co/C/Ta is around 1370 and 1055 Oe, respectively. The saturated *χ* (*χ*^sat^) standing for the largest SOT efficiency is around 10.0 and 8.3 Oe/(10^6^ A/cm^2^) for Pt/Co/Ta and Pt/Co/C/Ta, respectively. The decreased *χ*^sat^ for Pt/Co/C/Ta may be in that some interdiffusion and chemical reaction from the interface between Co and C as well as the interface between C and Ta increase the spin flipping probability and reduce the effective injection of spin current from the top Ta. In addition, the strength of the DMI exchange constant |*D*| can be also calculated from the measured |*H*_*DMI*_| using |*D*| = *μ*_0_*M*_*s*_*∆*|*H*_*DMI*_| [26], where *∆* is the DW width and it relates to exchange stiffness constant *A* and effective PMA energy density *K*_*eff*_ through *∆* = (*A*/*K*_*eff*_)^1/2^. Using *M*_*s*_ (respectively around 1.213×10^6^ and 1.288×10^6^ A/m for Pt/Co/Ta and Pt/Co/C/Ta) and *K*_*eff*_ (respectively around 4.1×10^5^ and 2.1×10^5^ J/m^3^ for Pt/Co/Ta and Pt/Co/C/Ta) as reported in the previous work and assuming *A* ≈ 1*.*5 × 10^−11^ J*/*m [27], we estimate |*D*| = 1.01 ± 0.16 mJ*/*m^2^ for Pt/Co/Ta and |*D*| = 1.15 ± 0.14 mJ*/*m^2^ for Pt/Co/C/Ta. The difference of |*D*| value seems to be weak in these two samples. This may be explained by that the total DMI strength results from the two contributions of bottom Pt/Co interfaces and top Co/Ta or Co/C interfaces. Since the bottom Pt/Co interfaces are very similar, they contribute equally to |*D*|. Whereas, for the contribution from the top Co/Ta or Co/C interface, Ma et al. [28] reported that |*D*| induced by Ta is much weaker than that by Pt. Hence, top Co/Ta interface is weak for the contribution of the total |*D*|. And the contribution from the top Co/C interface is also negligible due to the very weak spin-orbit coupling of C. It is also noted that both the bottom Pt/Co and top Co/Ta interfaces contribute to the DMI but may partially cancel each other [28], leading to the slightly decreased |*D*| for Pt/Co/Ta samples compared to Pt/Co/C/Ta samples. As a consequence, the similar |*D*| for Pt/Co/Ta and Pt/Co/C/Ta samples reveals that the DMI strength mainly comes from the contribution of the Pt/Co interface. Additionally, for these two samples, *H*_*DMI*_/*H*_*K*_ (respectively around 0.2 and 0.3 for Pt/Co/Ta and Pt/Co/C/Ta) is smaller than 2/π. While *H*_*DMI*_ is not beyond the theoretical threshold required to stabilize Néel DWs [25, 26], the chiral Néel DW in these two samples is demonstrated by observing the CIDWM behavior which will be discussed below. Meanwhile, the anomalous Hall loops under the in-plane bias field (*H*_*y*_) orthogonal the current direction are also investigated. Even though a large *H*_*y*_ is applied, the shift of *R*_*Hall*_-*H*_*z*_ loops is quite small (see the Additional file 1). It could be due to that *H*_*y*_ gradually transforms the chiral Néel-type DWs to Bloch-type DWs, and the effective field *H*_*SHE*_ is nearly zero for a Bloch-type DW according to the formula [10, 29, 30]:

1 $$ {\overset{\rightharpoonup }{H}}_{SHE}=-\frac{\mathrm{\hslash}{\theta}_{SHE}{J}_x}{2\left|e\right|{M}_s{t}_F}\left[\widehat{m}\times \left(\widehat{z}\times \widehat{j}\right)\right] $$

where, *θ*_*SHE*_, *Ms*, *t*_*F*_, *J*_*x*_, $\widehat{m}$ and $\widehat{j}$ represent the effective spin Hall angle, saturation magnetization of FM layer, thickness of FM layer, current density along *x* direction, unit vector of the magnetization and unit vector of the current density, respectively.

**Fig. 1:**
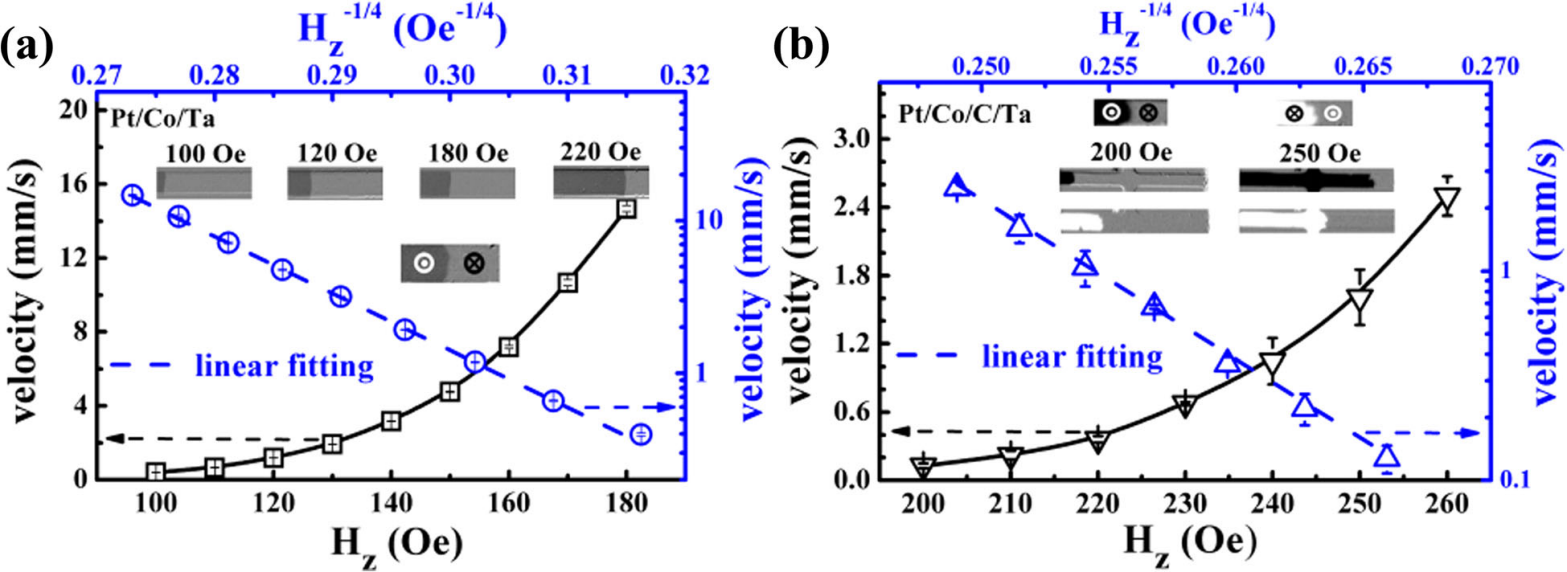
DW velocity as a function of out-of-plane field *H*_*z*_ for Pt/Co/Ta (**a**) and Pt/Co/C/Ta (**b**). The insets in **a** and **b** represent the snapshots of domains at different fields to show the DW shape

Next, the DW velocity (*v*) under the out-of-plane magnetic field and in-plane current pulse were measured using the Kerr microscope to investigate the DW motion behavior. A pre-prepared DW was formed using a magnetic field pulse just above the nucleation field after the racetrack saturated at an opposite large magnetic field. Velocity under *H*_*z*_ pulse is shown in Fig. 1a, b for two samples. For Pt/Co/C/Ta, *v* is still smaller than that of Pt/Co/Ta sample even under a large driving magnetic field. It is possibly due to the much more defects formation after C decoration, which increases the pinning fields [23]. It also can be seen that lg*v* is proportional to *H*_*z*_^-1/4^, indicating a creep regime of DW motion according to the creep law [31]:

2 $$ v={v}_0\exp \left[-\frac{U_c}{k_BT}{\left(\frac{H_{dep}}{H}\right)}^{1/4}\right] $$

where *U*_*C*_ is a characteristic energy related to the disorder-induced pinning potential, *k*_*B*_ is the Boltzmann constant, *T* is the temperature, and *H*_*dep*_ is the depinning field at which the Zeeman energy is equal to the DW pinning energy. The fitting slope gives a measurement of $\frac{U_c}{k_BT}{H_{dep}}^{1/4}=s$, *s* is around 37.4 and 76.5 Oe^1/4^ for Pt/Co/Ta and Pt/Co/C/Ta, respectively. Since the *H*_*dep*_ for Pt/Co/C/Ta is two times larger than that for Pt/Co/Ta [23], the difference of *H*_*dep*_^1/4^ between them is smaller than 1.5. However, the difference of *s* between them is larger than 2. It indicates that the Pt/Co/C/Ta sample has a larger pinning potential, which is consistent with the above discussion. Moreover, the insets in Fig. 1a, b also show the snapshots of domain images under different magnetic fields. One can see that the DW shape shows larger distribution for Pt/Co/C/Ta than that for Pt/Co/Ta. It also indicates that the pinning potential is not quite homogeneous in Pt/Co/C/Ta due to C decoration inducing randomly distributed pinning sites. Whereas, the regular DW tilting under the magnetic field is not observed for these two samples, which is different from the theoretical collective coordinate model [20].

**Fig. 2:**
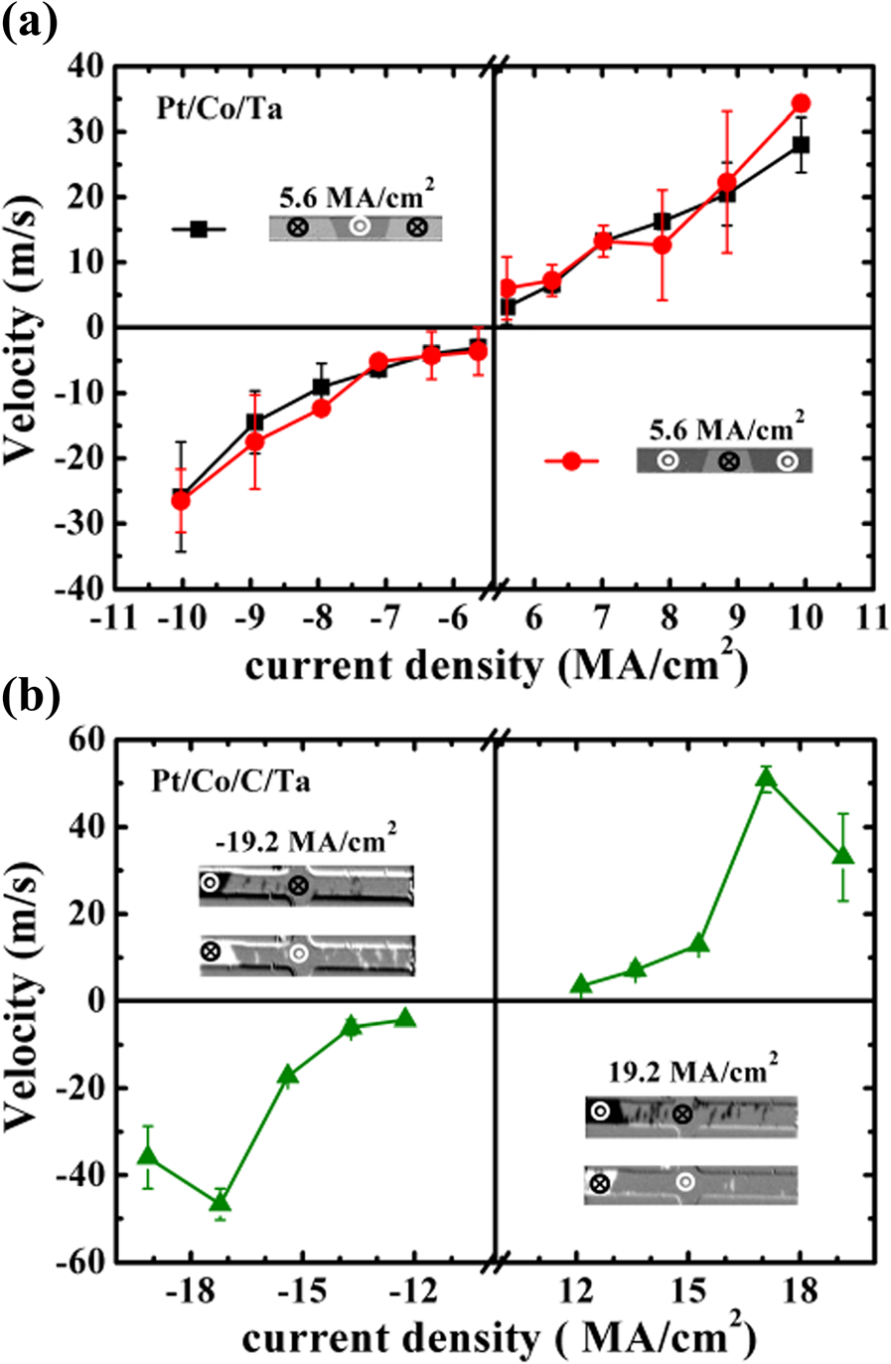
DW velocity against current density for Pt/Co/Ta (**a**) and Pt/Co/C/Ta (**b**). The insets in **a** and **b** represent the snapshots of the domain shape at the representative current density

**Fig. 3:**
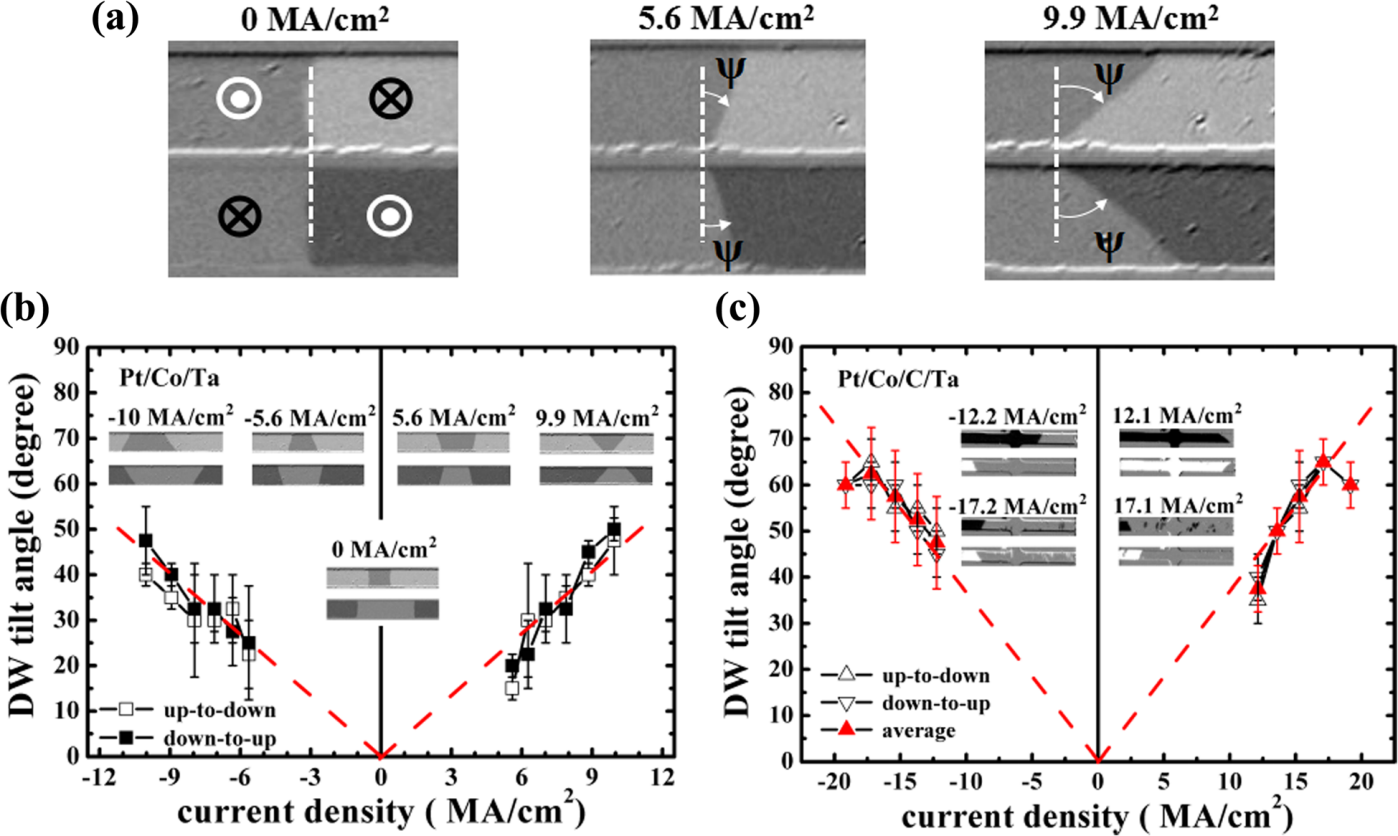
(**a**) Kerr images give the definition of DW tilt angle (*ψ*) and changes of *ψ* at different current densities from “up” to “down” state and “down” to “up” state, taking the Pt/Co/Ta sample as an example. DW tilt angle versus current density for Pt/Co/Ta (**b**) and Pt/Co/C/Ta (**c**). The insets in **b** and **c** represent the snapshots of the domain shape at different current densities

Afterwards, the CIDWM behavior was also investigated to make a comparison with field-induced DW motion. A up-to-down (U-D) or down-to-up (D-U) domain was firstly nucleated by a pulse magnetic field from a saturated state, and then, a pulse current was applied to push the DW motion using a pulse generator with pulse width in the range of 5–100 ns. Figure 2a, b shows the CIDWM velocity without any applied magnetic fields. The positive or negative velocity means the DW motion along or against the current direction. It implies the formation of a chiral Néel DW with the existence of DMI in these two samples [10, 30]. The increased velocity at the higher current density is due to the increased *H*_*SHE*_ acting on the chiral Néel DW. However, it needs double times larger current density to reach the same DW velocity in Pt/Co/C/Ta compared to that in Pt/Co/Ta structures. It may be ascribed to the decreased SOT efficiency and increased pinning potential barrier by C interfacial decoration. Moreover, the DW velocity by current driving is around 10^3^ times larger than that by magnetic field driving with the current generated effective field keeping the same value as magnetic field. It reveals that other mechanisms such as Joule heating and/or Oersted field generated from the current may also play a significant role in CIDWM. It should be noted, in Fig. 2b as well as Fig. 3c, that the decreases of DW velocity and DW tilt angle are observed in Pt/Co/C/Ta sample when the current density is at ± 19.2 MA/cm^2^. Meanwhile, one can see more nucleation area like white or black dots in the inset of Figs. 2b and 3c at higher current densities. This could be ascribed to that the thermal activates some random nucleation sites at large current densities due to the large Joule heating existing and the pinning potential barrier landscape could also be redistributed, which can exert an influence on the motion velocity and tilting angle.

**Fig. 4:**
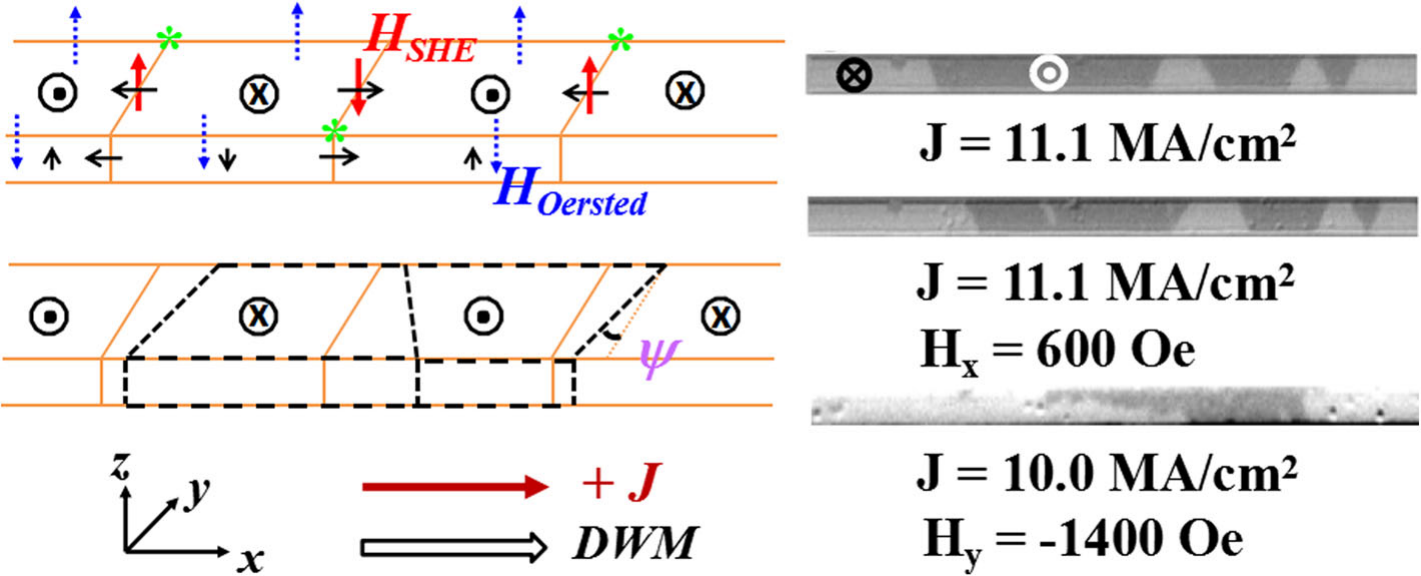
Schematic DW motion and domain shapes at a current density *J*. The left upper panel shows the domain with U-D-U-D sketches and the magnetization orientation (thin black arrow) in domain and DWs. Once a current applied, the generated *H*_*SHE*_ acting on the DWs are shown as red thick arrows, while the Oersted fields (*H*_*Oersted*_) at both sides of the racetrack are shown as dash blue arrows. The left lower panel shows the corresponding change of the domain shape (denoted as dash thick black blocks) under the action of *H*_*SHE*_ and *H*_*Oersted*_. The right panel shows the effect of in-plane magnetic fields on the domain shape for Pt/Co/Ta

During the current-induced DW motion, DW tilting phenomenon is clearly observed in these two samples and the tilting is gradually formed in a time-dependent observation with a sufficient short pulse as the driving force. In order to gain an insight into the current-induced DW tilting, we measure DW tilt angle (*ψ*) which is defined in Fig. 3a at different current densities. It should also be noted that the tilt angle may slightly change during the motion due to the wide depinning distribution along the racetracks, which will result in a large measuring error at a specific current density. From Fig. 3b, c one can see a roughly linear dependence of the tilt angle on the current density for both samples. It agrees with the previous theoretical work [20], in which approximately linear dependences of the tilt angle and DW velocity on the lower current density can be observed. However, a large DW tilt angle happens with at least one order larger current density in their simulation. This is not consistent with our observation and the tilting behavior is also not observed during the field-induced DW motion in our experiment. Therefore, the influence of the DMI or graded distribution of the pinning potential barrier on the current-induced DW tilting can be weak. Besides, the anomalous Hall effect may also lead to the DW tilting, but the contribution is expected to be small in a nanometer-thick racetrack [20]. One possible explanation is that the applied current not only generates spin Hall effective field *H*_*SHE*_, but also the Oersted field (*H*_*Oersted*_) which also can lead to DW motion. Both *H*_*SHE*_ and *H*_*Oersted*_ could have an influence on the DW tilting. In Fig. 4, we plot the sketch of these effective fields to clarify the DW tilting behavior. The domain arrangement is shown as U-D-U-D sketches, and the magnetization in the DW with a left-handed chirality is shown as thin black arrow along the in-plane orientation. In a thin uniform racetrack, if the thickness (*t*) is much less than the width (*w*), the generated *H*_*Oersted*_ is concentrated on the two edges and its averaged component over the thickness can be calculated by *H*_*Oersted*_ *=* ±*jt*[3 + 2ln*w*/*t*]/4π [22]. The obtained *H*_*Oersted*_ are around 19.6 and 37.4 Oe for Pt/Co/Ta and Pt/Co/C/Ta using the maximum current density of 10.0 and 19.2 MA/cm^2^, respectively, which is comparable to the spin Hall effective field *H*_*SHE*_ (around 100.0 and 159.4 Oe for Pt/Co/Ta and Pt/Co/C/Ta at the same current densities). Since *H*_*SHE*_ and *H*_*Oersted*_ have the same direction at the positions marked as green stars, a larger effective field will act on the DW at the green star area, which results in a much larger velocity compared to that at the opposite area of the green stars in the racetrack. Therefore, a tilting DW with a specific trapezoid shape can be formed shown in the left lower panel of Fig. 4. The inset of Fig. 3b for Pt/Co/Ta also obviously shows the similar shape at some representative current densities. Moreover, the increased tilt angle at the higher current density may be explained by the large velocity difference in both edges of the racetrack due to the increased *H*_*Oersted*_. Meanwhile, one can find that the domain shape will have a transformation once the domain arrangements and/or the current polarity changes according to the above analysis. All the sketched domain shape at a current pulse is consistent with the experimental observations. Moreover, the above explanation about DW tilting is also valid when an in-plane *H*_*x*_ or *H*_*y*_ is applied. When *H*_*x*_ is applied, it will change the magnetization orientation in DWs. Therefore, the *H*_*SHE*_ will change a sign for the DW with its original horizontal magnetization opposite to *H*_*x*_, which makes the trapezoid-shape domain expand or shrink (depending on the sign of *H*_*x*_) as shown in the right middle panel of Fig. 4. When *H*_*y*_ is applied, a strong *H*_*y*_ will change a Néel-type DW to a Bloch one. *H*_*SHE*_ will become zero according to Eq. (1), and only current generated Oersted field *H*_*Oersted*_ drives the DW motion. This will make the domain expand at one edge. One can also see that the change of the domain at the edge happens as *H*_*y*_ is around − 1400 Oe as shown in the right lower panel of Fig. 4. This is consistent with the analysis that *H*_*Oersted*_ as the only driving force is responsible for the DW motion. However, it cannot lead to a regular DW tilting behavior. Therefore, the current-induced DW tilting could be ascribed to the current-induced Oersted field combined with spin Hall effective field.

## Conclusions

In summary, current-induced domain wall motion and tilting are observed in Pt/Co/Ta and Pt/Co/C/Ta structures. The DMI strength and SOT efficiency are obtained using a transport measurement method which can reach to 1.01 ± 0.16 (1.15 ± 0.14) mJ/m^2^ and 10.0 (8.3) Oe/MA/cm^2^ for Pt/Co/Ta (Pt/Co/C/Ta) samples, respectively. The similar DMI strength for Pt/Co/Ta and Pt/Co/C/Ta samples reveals that the DMI strength mainly comes from the contribution of the Pt/Co interface. The reduced DW velocity in field-induced DW motion for Pt/Co/C/Ta indicates the DW velocity is related to the pinning potential barrier. In addition, current-generated Joule heating and Oersted field play a significant role in the DW motion and tilting. For the racetrack memory application, the large current-generated Oersted field should be considered since it will dramatically change the recording bit shape and even shrink the area of the recording bits. This may be not beneficial to the practical application. Our findings could provide some designing prospects to drive DW motion in SOT-based racetrack memories.

## Additional file

Additional file 1: The estimation of the DMI effective fields and SOT efficiency as well as the influence of an in-plane bias field *H*_*x*_ or *H*_*y*_ on the anomalous Hall loops. (DOCX 529 kb)

## Abbreviations

CIDWM: Current-induced domain wall motion
DMI: Dzyaloshinskii–Moriya interaction
D-U: Down-to-up
DW: Domain wall
FIDWM: Field-induced domain wall motion
FMs: Ferromagnets
HM: Heavy metal
PMA: Perpendicular magnetic anisotropy
SOT: Spin-orbit torque
STT: Spin transfer torque
U-D: Up-to-down
