# Supplementary material for: Current-Induced Domain Wall Motion and Tilting in Perpendicularly Magnetized Racetracks
Source: Nanoscale Res Lett. 2018 Aug 15;13:238. doi: 10.1186/s11671-018-2655-6 (PMC6093834; doi:10.1186/s11671-018-2655-6)
Supplement: Supplementary file 1 — The estimation of the DMI effective fields and SOT efficiency as well as the influence of an in-plane bias field Hx or Hy on the anomalous Hall loops. (DOCX 529 kb) [file 11671_2018_2655_MOESM1_ESM.docx]

**Current-induced domain wall motion and tilting in perpendicularly magnetized racetracks**

**Dong Li**^1,2^**, Baoshan Cui**^1^**, Jijun Yun**^1^**, Minzhang Chen**^1^**, Xiaobin Guo**^1^**, Kai Wu**^1^**, Xu Zhang**^1^**, Yupei Wang**^1^**, Jian Mao**^1^**, Yalu Zuo**^1^**, Jianbo Wang**^1^**, Li Xi**^[[1]](#footnote-1)^*****

**Additional file 1**

**Contents:**

**S1.** **The influence of an in-plane bias field *H_x_* on the anomalous Hall resistance loops (*R_Hall_*-*H_z_*) and switching field *vs* current density (*H_SW_-J*), as well as SOT efficiency *χ* as a function of *H_x_.***

**S2. The influence of an in-plane bias field *H_y_* on the anomalous Hall resistance loops (*R_Hall_*-*H_z_*) and switching field *vs* current density (*H_SW_-J*), as well as SOT efficiency *χ* as a function of *H_y_.***

**S1.** **The influence of an in-plane bias field *H_x_* on the anomalous Hall resistance loops (*R_Hall_*-*H_z_*) and switching field *vs* current density (*H_SW_-J*), as well as SOT efficiency *χ* as a function of *H_x_.***

**
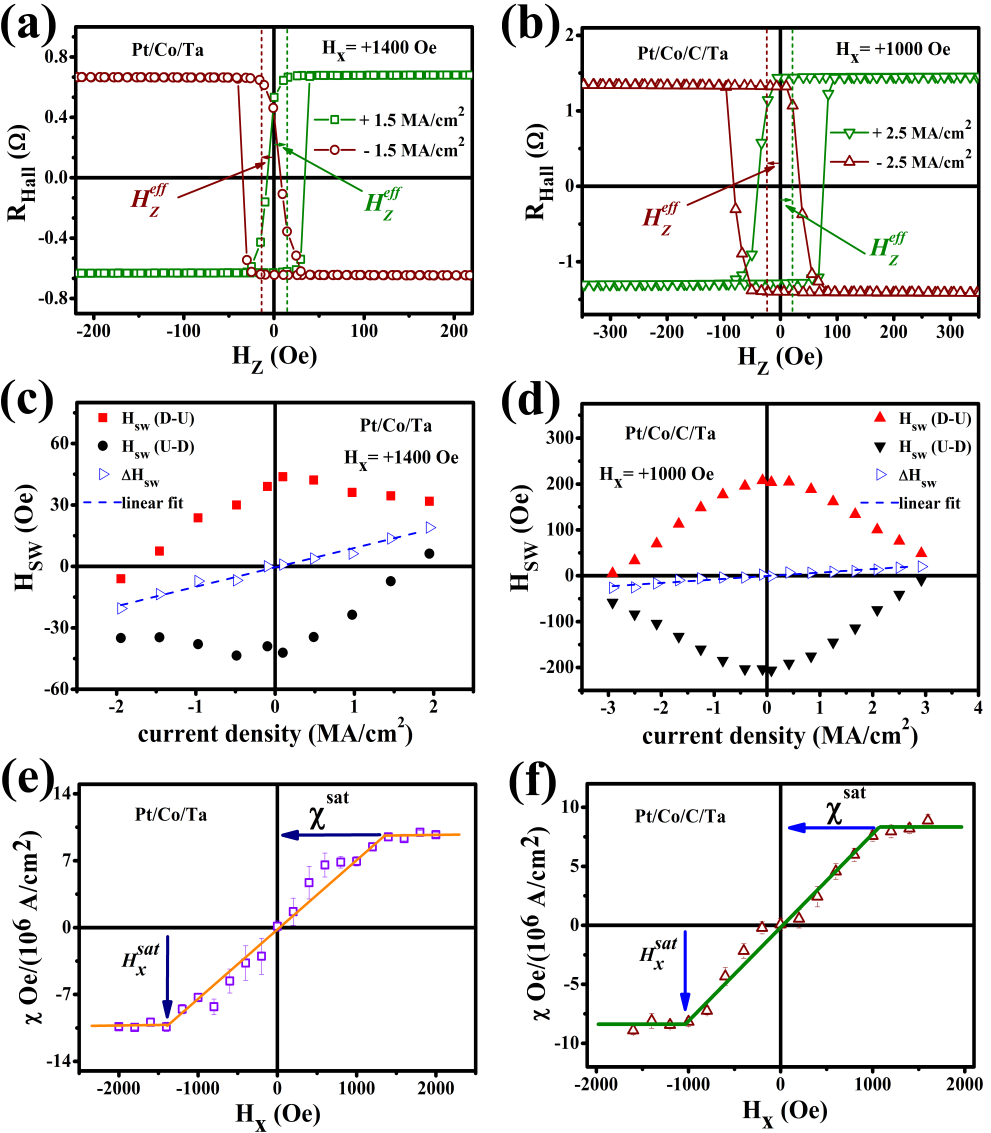
**

**Figure S1.** Representative anomalous Hall resistance loops with the current density of ± 1.5 MA/cm^2^ and in-plane bias field *H_x_* = +1400 Oe for a Pt/Co/Ta sample **(a)** , and with current density of ± 2.5 MA/cm^2^ and in-plane bias field *H_x_* = +1000 Oe for a Pt/Co/C/Ta sample **(b)**. *Heff Z* represents the center of the hysteresis loops. Switching (depinning) fields *H_SW_* for down-to-up and up-to-down magnetization reversals versus current density, with *H_x_* = +1400 Oe for Pt/Co/Ta **(c)** and with *H_x_* = +1000 Oe for Pt/Co/C/Ta **(d)**. The dash lines represent linear fitting to ∆*H_SW_* data. The measured effective *χ* as a function of the applied in-plane bias field *H_x_* for Pt/Co/Ta **(e)** and Pt/Co/C/Ta **(f)**.

The representative shifted anomalous Hall resistance loops under the applied in-plane bias field *H_x_* along the current direction are shown in Figure S1 (a) and (b). The horizontal shift of hysteresis loops corresponds to the contribution from the current induced SOT effective field, which is deduced from the up-down (U-D) and down-up (D−U) magnetization switching fields (*H_SW_*) as *Heff Z*= (*HU-D SW*+ *HD-U SW*)/2. The DMI will induce a chiral Néel domain wall (DW) once nucleation happened in the demagnetization process, and applying an in-plane magnetic field will change the magnetization in the DW towards the in-plane magnetic field direction. When a large current passing through the Pt and Ta layers, the generated spin currents with the same orientation of electron spin between the two sides of Co will generate an out-of-plane spin Hall effective field (*H_SHE_*). The direction of *H_SHE_* is related to the current direction and magnetization orientation in the DW according to the formula: [1, 2, 3]

 (S1)

where, *θ_SHE_*, *Ms*, *t_F_*, *J_x_*,$\hat{m}$and$\hat{j}$represent the effective spin Hall angle, saturation magnetization of the FM layer, thickness of the FM layer, current density along *x* direction, unit vector of the magnetization and unit vector of the current density, respectively. Thus, U-D and D−U switching fields will be written as *HD-U SW* = *Heff Z* (I_dc_) + *H_c_* (I^2^_dc_) and *HU-D SW* = *Heff Z* (I_dc_) − *H_c_* (I^2^_dc_). Using the above definition, the Joule heating contribution can be eliminated by only considering the horizontal shift of the hysteresis loop center as discussed in the literature [4]. *H_SW_* under a large in-plane magnetic field *H_x_* with the variation of *J* for these two samples are shown in Figure S1(c) and (d). One can see that *H_SW_* for U-D and D−U states are asymmetric, which is caused by the current-induced *Heff Z*. *ΔH_sw_* (defined as (*HU-D SW*+ *HD-U SW*)/2, that is *Heff Z*) is almost linear with the current density for Pt/Co/Ta and Pt/Co/C/Ta and the fitting slope determines the SOT efficiency *χ*. Figure S1(e) and (f) show the SOT efficiency *χ* under the different *H_x_* for the two samples. The value of *χ* under a specific *H_x_* is determined by the magnetization orientation in the DW with the competition between the inherent DMI effective field (*H_DMI_*) and applied in-plane bias field *H_x_*. The saturation magnetic field for Pt/Co/Ta and Pt/Co/C/Ta is respectively around 1370 and 1055 Oe, which roughly gives a measure of *H_DMI_*. The saturated *χ* (*χ^sat^*) stands for the largest SOT efficiency. *χ^sat^* is around 10.0 and 8.3 Oe /(10^6^ A/cm^2^) for Pt/Co/Ta and Pt/Co/C/Ta.

**S2. The influence of an in-plane bias field *H_y_* on the anomalous Hall resistance loops (*R_Hall_*-*H_z_*) and switching field *vs* current density (*H_SW_-J*), as well as SOT efficiency *χ* as a function of *H_y_.***


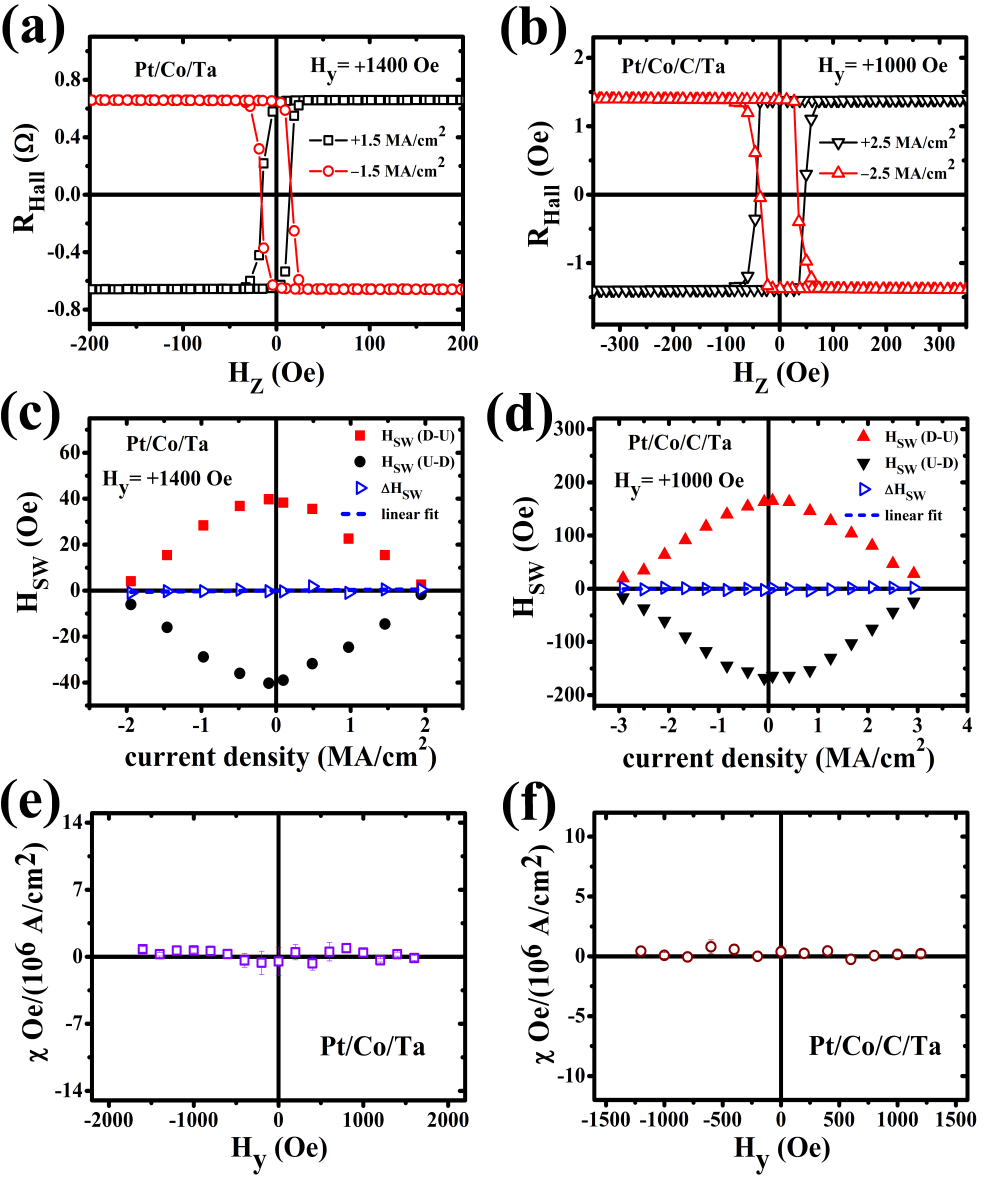


**Figure S2.** The anomalous Hall resistance loops for **(a)** Pt/Co/Ta with the current density *J* = ±1.5 MA/cm^2^ and an in-plane bias field *H_y_* = +1400 Oe; **(b)** Pt/Co/C/Ta with the current density *J* = ±2.5 MA/cm^2^ and an in-plane bias field *H_y_* = +1000 Oe. The switching fields *H_sw_* for down-to-up (D-U) and up-to-down (U-D) magnetization reversals as a function of the current density, with *H_y_* = +1400 Oe for Pt/Co/Ta **(c)** and *H_y_* = +1000 Oe for Pt/Co/C/Ta **(d)**. The dash lines represent the linear fitting to *ΔH_sw_*. The measured SOT efficiency *χ* versus applied in-plane field *H_y_* for Pt/Co/Ta **(e)** and Pt/Co/C/Ta **(f)**.

In order to investigate the influence of an in-plane bias field *H_y_* on the anomalous Hall resistance loops, we measure the *R_Hall_*-*H_z_* loops for Pt/Co/Ta with the current density *J* = ±1.5 MA/cm^2^ and in-plane bias field *H_y_* = +1400 Oe as well as Pt/Co/C/Ta with the current density *J* = ±2.5 MA/cm^2^ and in-plane bias field *H_y_* = +1000 Oe shown in Figure S2 (a) and (b), respectively. As expected, no shifts are observed for both samples, indicating that *H_y_* has a trend to change the chiral Néel-type DW to the Bloch-type DW, and the Slonczewski-like spin Hall effective field *H_SHE_* is nearly zero for a Bloch DW according to Eq. (S1). In addition, we also obtain switching fields *H_sw_* for down-to-up (D-U) and up-to-down (U-D) magnetization reversals as a function of the current density by measuring a series of *R_Hall_*-*H_z_* loops at different currents and in-plane fields *H_y_*. As shown in Figure S2 (c) and (d), *ΔH_sw_* is almost constant with the current density and the linear fitting is the curve with the slope of zero for both Pt/Co/Ta and Pt/Co/C/Ta, which also implying that the value of *H_SHE_* is quite small under the in-plane bias field *H_y_*. Finally, we summary the SOT efficiency *χ* as a function of the applied in-plane field *H_y_* for Pt/Co/Ta and Pt/Co/C/Ta. From Figure S2 (e) and (f), one can see that *χ* nearly keeps invariant and is quite small, indicating that the in-plane field *H_y_* makes no contribution to the Slonczewski-like spin Hall effective field *H_SHE_* and DMI effective field *H_DMI_*.

**References**

1. Emori S, Bauer U, Ahn SM, Martinez E, Beach GSD (2013) Current-driven dynamics of chiral ferromagnetic domain walls. Nat Mater 12**:**611
2. Khvalkovskiy AV, Cros V, Apalkov D, Nikitin V, Krounbi M, Zvezdin KA, Anane A, Grollier J, Fert A (2013) Matching domain-wall configuration and spin-orbit torques for efficient domain-wall motion. Phys Rev B 87:020402
3. Yu GQ, Upadhyaya P, Wong KL, Jiang WJ, Alzate JG, Tang JS, Amiri PK, Wang KL (2014) Magnetization switching through spin-Hall-effect-induced chiral domain wall propagation. Phys Rev B 89:104421
4. Pai CF, Mann M, Tan AJ, Beach GSD (2016) Determination of spin torque efficiencies in heterostructures with perpendicular magnetic anisotropy. Phys Rev B 93:144409

1. ^*^Correspondence: [xili@lzu.edu.cn](mailto:xili@lzu.edu.cn)

   ^1^Key Laboratory for Magnetism and Magnetic Materials of Ministry of Education & School of Physical Science and Technology, Lanzhou University, Lanzhou 730000, People’s Republic of China

   ^2^Research Institute of Materials Science, Shanxi Normal University, Linfen 041004, People’s Republic of China [↑](#footnote-ref-1)
